# Chemokine Receptor 5 Antagonism Causes Reduction in Joint Inflammation in a Collagen-Induced Arthritis Mouse Model

**DOI:** 10.3390/molecules26071839

**Published:** 2021-03-25

**Authors:** Mushtaq A. Ansari, Ahmed Nadeem, Saleh A. Bakheet, Sabry M. Attia, Mudassar Shahid, Faris S. Alyousef, Mohammed A. Alswailem, Mohammed Alqinyah, Sheikh F. Ahmad

**Affiliations:** 1 Department of Pharmacology and Toxicology, College of Pharmacy, King Saud University, Riyadh 11451, Saudi Arabia; muansari@ksu.edu.sa (M.A.A.); anadeem@ksu.edu.sa (A.N.); sbakheet@ksu.edu.sa (S.A.B.); attiasm@ksu.edu.sa (S.M.A.); m-alswailem@outlook.com (F.S.A.); f-s-y18@hotmail.com (M.A.A.); malqinyah@ksu.edu.sa (M.A.); 2 Department of Pharmacology and Toxicology, Faculty of Pharmacy, Al-Azhar University, Nasr City, Cairo 11884, Egypt; 3 Department of Pharmaceutics, College of Pharmacy, King Saud University, Riyadh 11451, Saudi Arabia; mahmad1@ksu.edu.sa

**Keywords:** maraviroc, CCR5 antagonist, inflammation, collagen-induced arthritis, CD8 cells

## Abstract

Rheumatoid arthritis (RA) is a chronic inflammatory disease mainly affecting the synovial joints. A highly potent antagonist of C-C chemokine receptor 5 (CCR5), maraviroc (MVC), plays an essential role in treating several infectious diseases but has not yet been evaluated for its potential effects on RA development. This study focused on evaluating the therapeutic potential of MVC on collagen-induced arthritis (CIA) in DBA/1J mice. Following CIA induction, animals were treated intraperitoneally with MVC (50 mg/kg) daily from day 21 until day 35 and evaluated for clinical score and histopathological changes in arthritic inflammation. We further investigated the effect of MVC on Th9 (IL-9, IRF-4, and GATA3) and Th17 (IL-21R, IL-17A, and RORγT) cells, TNF-α, and RANTES in CD8+ T cells in the spleen using flow cytometry. We also assessed the effect of MVC on mRNA and protein levels of IL-9, IL-17A, RORγT, and GATA3 in knee tissues using RT-PCR and western blot analysis. MVC treatment in CIA mice attenuated the clinical and histological severity of inflammatory arthritis, and it substantially decreased IL-9, IRF4, IL-21R, IL-17A, RORγT, TNF-α, and RANTES production but increased GATA3 production in CD8+ T cells. We further observed that MVC treatment decreased IL-9, IL-17A, and RORγt mRNA and protein levels and increased those of GATA3. This study elucidates the capacity of MVC to ameliorate the clinical and histological signs of CIA by reducing pro-inflammatory responses, suggesting that MVC may have novel therapeutic uses in the treatment of RA.

## 1. Introduction

Rheumatoid arthritis (RA) affects about 1% of the global population [1]. The etiology of RA involves a combination of genetic and environmental factors, although the pathogenesis of RA remains unclear. RA is a progressive immune-mediated inflammatory disease characterized by inflammatory cell infiltration and structural damage in affected joints [2,3]. Dysregulation of protective immune responses causes autoimmune diseases. T cell signaling has been shown to play a critical role in RA progression [4]. Moreover, both the innate and adaptive immune systems are also involved in RA development and pathogenesis [5]. It has further been reported that during the development of RA, increased production of adhesion molecules and chemokine receptors leads to the destruction of cartilage and bone [1].

Interleukin (IL)-9 has been recently described as characteristic of Th9 cells and is associated with several inflammatory conditions, such as neoplastic, infectious, and autoimmune diseases [6,7]. Recently, the potential roles of IL-9 and Th9 cells have been reported in the peripheral blood, synovial tissues, and fluid of RA patients [8], and one study suggested the functional role of IL-9 in the synovial fluid of RA patients [9]. A recent study has demonstrated that interferon regulatory factor 4 (IRF4), a transcription factor regulating IL-17 and IL-21 production, is involved in autoimmunity development [10]. One study showed that increased GATA-binding protein 3 (GATA3) expression protects against joint inflammation and also reduces Th17 cell differentiation during experimental arthritis [11].

Pro-inflammatory cytokines such as IL-17A are considered as crucial players in RA pathogenesis [12]. An elevated frequency of IL-17A-producing Th17 cells has been reported in RA [13]. IL-17A in synovial fluids from patients with RA is a potent stimulator of osteoclastogenesis and bone resorption [14]. RAR-related orphan receptor γt (RORγt), a master transcription factor, directs the differentiation program of pro-inflammatory Th17 cells [15]. It was further reported that IL-21R was expressed on multiple cell types, including T and B cells from peripheral blood and synovial fluid [16]. The cytokine TNF-α plays a significant role in modulating immune response, and its level is increased in synovial tissues of CIA mice [17]. Regulated on activation, normal T cell expressed and secreted (RANTES) is a chemoattractant for monocytes and T cells, and its expression plays an important role in RA pathogenesis [18].

Maraviroc (MVC), a potent and selective small-molecule inhibitor of C-C chemokine receptor 5 (CCR5), displays therapeutic efficacy against HIV infection [19,20]. In addition, by blocking the signaling of CCR5 ligands, MVC administration effectively inhibits the migration and effector functions of CCR5-bearing leukocytes exerting immunomodulatory and anti-inflammatory effects [21,22]. Previous data also showed that MVC reduced the number of T cells and percentages of Th1 and Th17 cells and suppressed dendritic cell maturation [23]. A recent study showed that MVC administration inhibited leukocyte trafficking and mucosal inflammation [24]. The development of new RA treatments depends on animal models, and RA pathogenesis is clearly observable using a mouse model of collagen-induced arthritis (CIA). Despite the progress achieved in RA treatment in recent decades, alternative therapies with high efficacy are required to treat RA. In the present study, we explored the role of MVC in a CIA mouse model. Furthermore, the molecular mechanisms of MVC were further explored by examining RORγT/IRF4 transcription factor signaling in CIA mice.

## 2. Results

### 2.1. MVC Exerts Therapeutic Effects in CIA Mice

**Figure 1:**
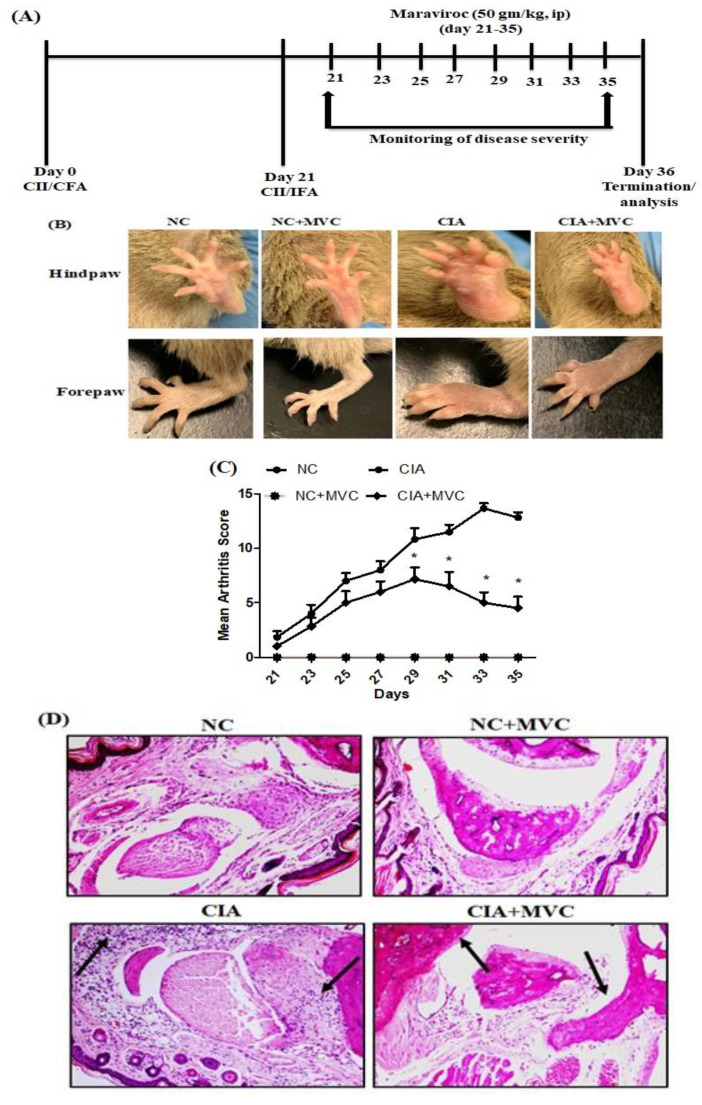
MVC treatment prevents inflammation in collagen-induced arthritis (CIA) mice. DBA/1J mice were first immunized via intradermal injection of bovine type II collagen emulsified in complete Freund’s adjuvant. The second immunization was administered 21 days later in incomplete Freund’s adjuvant. (**A**) Work flow of MVC treatment for CIA in mice. Briefly, mice were first immunized with injection (s.c.) of emulsion containing bovine type II collagen (CII)/CFA on day 0 and were subsequently immunized with emulsion containing CII/IFA on day 21. Mice were treated with MVC from day 21 to day 35. The CIA mouse model was established, treated with MVC, and evaluated for clinical parameters of arthritis, including joint swelling (**B**), and mean arthritis score in CIA mice (**C**). Histological analysis of the joints showed a significant improvement in inflammation with less damage to the joint space in MVC-treated mice (**D**). Normal control (NC) mice received 1% (*v*/*v*) DMSO in saline and MVC (50 mg/kg) intraperitoneally (ip) daily from days 21 to 35. CIA mice were treated with MVC (50 mg/kg) ip after the second immunization. The level of significance was set at * *p* < 0.05 compared with the CIA control group. Data are presented as mean ± SD (n = 6).

To investigate whether MVC could prevent CIA development, MVC was administered from day 21 to 35, when signs of arthritis were observed. After CIA onset, arthritis scores were used to determine the clinical severity of arthritis inflammation. The arthritis scores continuously increased in CIA control mice, whereas it significantly decreased in MVC-treated mice (Figure 1B,C, photos of the hindpaw and forepaw of one mouse from each group are shown). In order to further study, the effect of MVC on CIA mice, histological sections of the knee joints were evaluated by H&E staining (Figure 1D). The knee joints of CIA mice revealed severe inflammatory signs such as synovial hyperplasia, inflammatory cell infiltration, bone erosions, and pannus formation, whereas MVC treatment improved these pathological changes, reduced the infiltration and ameliorated the severity of bone erosions (Figure 1D). Our results showed the therapeutic effect of MVC in CIA mice.

### 2.2. Effect of MVC on Th9-Related Transcription Factors

**Figure 2:**
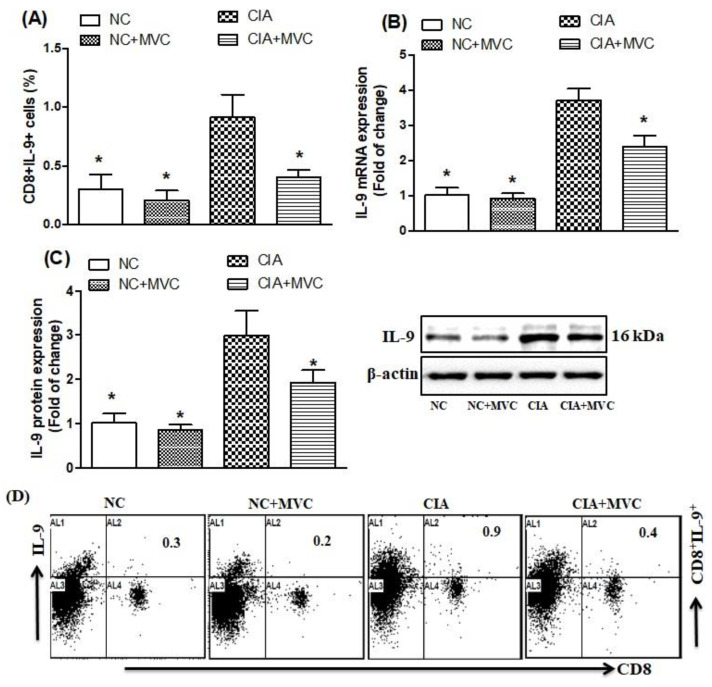
(**A**) The effect of MVC on IL-9-producing CD8+ T cells was analyzed through flow cytometry in the spleen. (**B**,**C**) The expression levels of IL-9 mRNA (**B**) and protein (**C**) were analyzed by RT-PCR and western blot, respectively, in knee tissues from MVC-treated mice. (**D**) Representative dot plots of one mouse from each group. Normal control (NC) mice received 1% (*v*/*v*) DMSO in saline and MVC (50 mg/kg) intraperitoneally (ip) daily from days 21 to 35. CIA mice were treated with MVC (50 mg/kg) ip after the second immunization. The level of significance was set at * *p* < 0.05 compared with the CIA control group. Data are presented as mean ± SD (n = 6).

We further determined whether MVC administration could inhibit IL-9-producing Th9 cells and related transcription factors in CIA mice. We found that the number of IL-9-producing CD8+ T cells significantly increased in spleens of CIA control mice compared with NC mice (Figure 2A,D; as shown in Figure 2D, the representative dot plots of one mouse from each group). Compared to the CIA control, the number of IL-9-producing CD8+ T cells was markedly reduced in MVC-treated CIA mice (Figure 2A,D). To further clarify the mechanism of MVC, we used RT-PCR and western blotting to examine changes in mRNA and protein levels of IL-9 in knee tissues. There were statistically significant differences in mRNA and protein expression in the knee tissues of CIA control mice or MVC-treated mice (Figure 2B,C). Taken together, the therapeutic effect of MVC resulted from IL-9 cytokine suppression.

**Figure 3:**
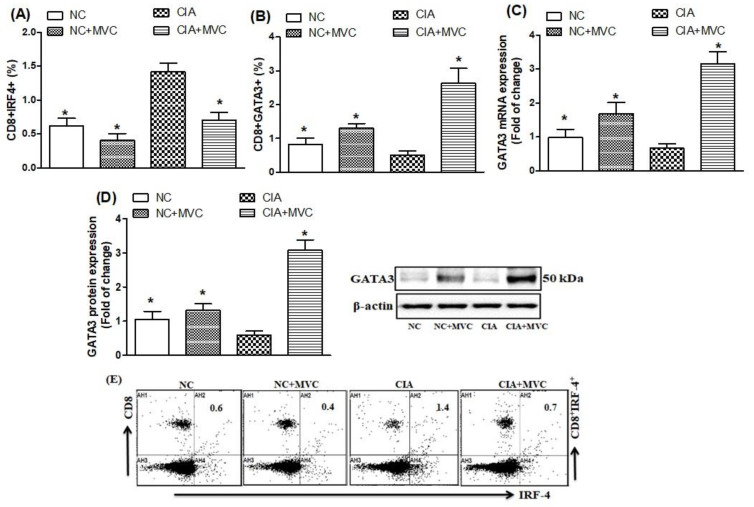
(**A**,**B**) The effects of MVC on IRF4- and GATA3-producing CD8+ T cells were analyzed through flow cytometry in the spleen. The expression levels of GATA3 mRNA (**C**) and protein (**D**) were analyzed by RT-PCR and western blot, respectively, in knee tissues from MVC-treated mice. (**E**) Representative dot plots of one mouse from each group. Normal control (NC) mice received 1% (*v*/*v*) DMSO in saline and MVC (50 mg/kg) intraperitoneally (ip) daily from days 21 to 35. CIA mice were treated with MVC (50 mg/kg) ip after the second immunization. The level of significance was set at * *p* < 0.05 compared with the CIA control group. Data are presented as mean ± SD (n = 6).

We further investigated the effects of MVC administration on Th9 related transcription factors, i.e., IRF4 and GATA3. As shown in Figure 3A,E, higher levels of IRF4-producing CD8+ T cells were observed in the spleens of CIA control mice than in normal mice. MVC treatment in CIA mice significantly decreased IRF4-producing CD8+ T cells (Figure 3A,E; as shown in Figure 3E, the representative dot plots of one mouse from each group) and caused a significant increase in the level of GATA3-producing CD8+ T cells compared with CIA control mice (Figure 3B). MVC treatment in CIA mice also increased both mRNA (Figure 3C) and protein levels (Figure 3D) of GATA3 in knee tissues. Our results indicate that MVC administration could attenuate RA progression through downregulating IRF4 and upregulating GATA3 expression in a CIA mouse model.

### 2.3. MVC Treatment Inhibits Th17 Cell Related Signaling

**Figure 4:**
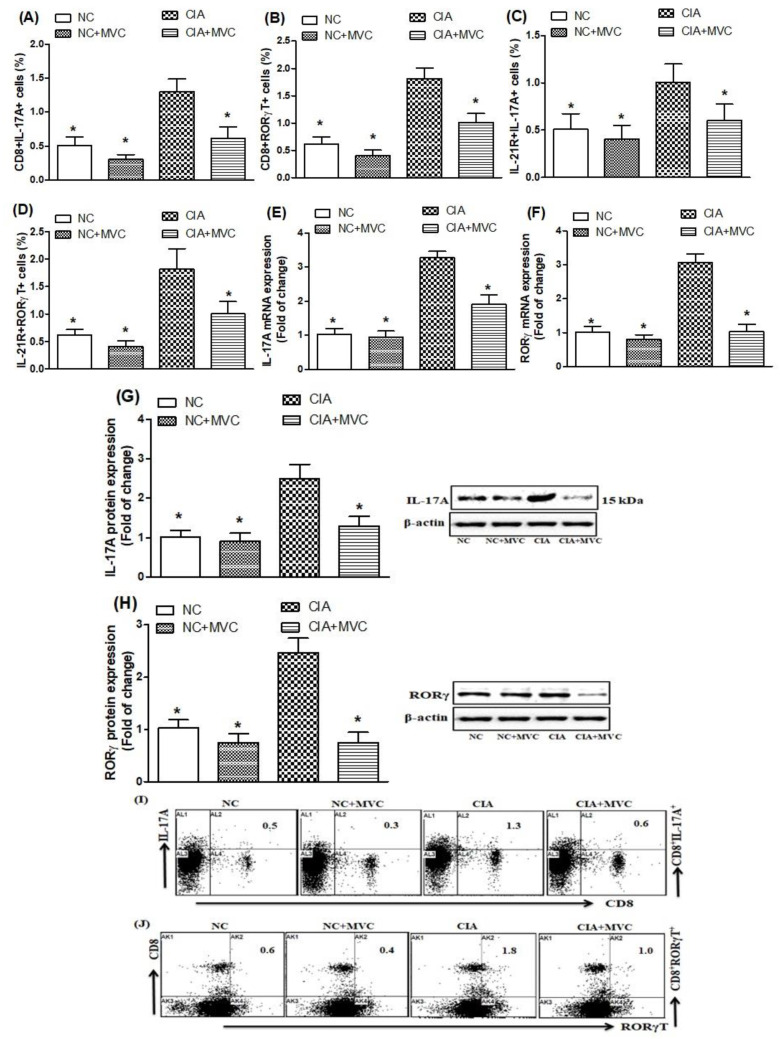
(**A**–**D**). The effects of MVC on IL-17A- and RORγt-producing CD8+ T cells and IL-21+ receptor were analyzed through flow cytometry in the spleen. The mRNA expression levels of IL-17A (**E**) and RORγt (**F**) were analyzed by RT-PCR in knee tissues from MVC-treated mice. The protein expression levels of IL-17A (**G**) and RORγt (**H**) were analyzed by western blot in knee tissues from MVC-treated mice. (**I**,**J**) Representative dot plots of one mouse from each group. Normal control (NC) mice received 1% (*v*/*v*) DMSO in saline and MVC (50 mg/kg) intraperitoneally (ip) daily from days 21 to 35. CIA mice were treated with MVC (50 mg/kg) ip after the second immunization. The level of significance was set at * *p* < 0.05 compared with the CIA control group. Data are presented as mean ± SD (n = 6).

Our next objective was to investigate whether MVC can modulate Th17 cells-related signaling as these play an important role in joint inflammation. Flow cytometry analysis was carried out to examine the effect of MVC in CIA mice. We investigated whether MVC treatment affected IL-17A- and RORγt-producing CD8+ T cells in the spleen. Our data show that these cells were significantly increased in the spleens of CIA control mice compared with saline-treated mice (Figure 4A,B,I,J; as shown in Figure 4I,J, the representative dot plots of one mouse from each group). Interestingly, MVC treatment of CIA mice significantly decreased the number of CD8+IL-17A+ and CD8+RORγt+ cells (Figure 4A,B,I,J). 

We also examined the effect of MVC treatment on IL-17A- and RORγT-producing IL-21R+ cells in the spleen. MVC-treated CIA mice had significantly decreased IL-17A- and RORγT-producing IL-21R+ cells compared to the CIA control mice (Figure 4C,D). We further examined the effect of MVC treatment on IL-17A and RORγt mRNA and protein levels in the knee tissues and found that each were reduced in the knee tissues of MVC-treated CIA mice compared with those of CIA control mice (Figure 4E–H). These results suggest that MVC treatment exerts its therapeutic effect through downregulation of Th17 cell signaling during joint inflammation in our CIA model.

### 2.4. MVC Treatment Inhibits TNF-α and RANTES Production in CIA Mice

**Figure 5:**
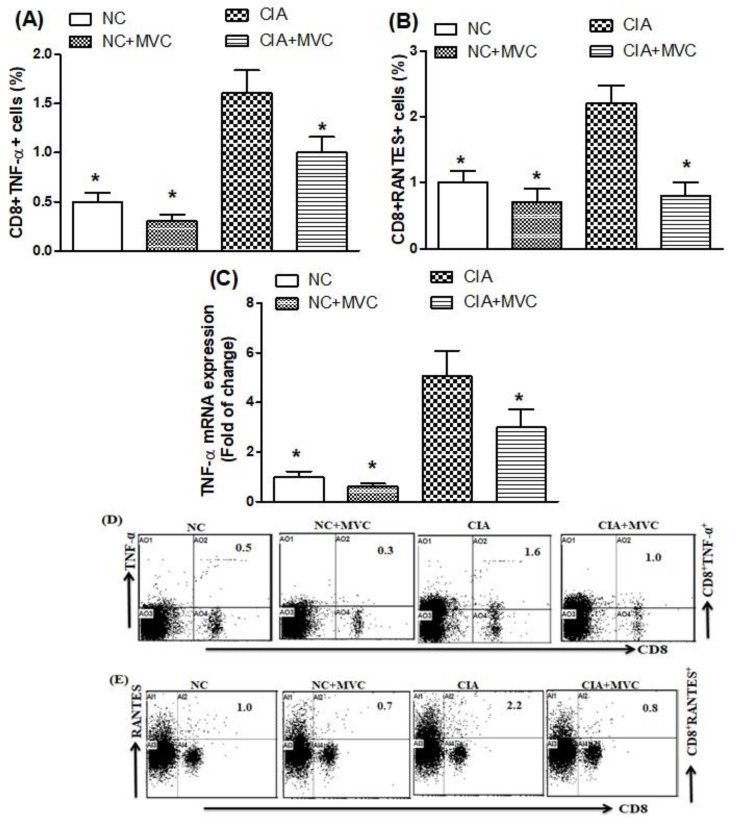
(**A**,**B**). The effects of MVC on TNF-α- and RANTES-producing CD8+ T cells were analyzed through flow cytometry in the spleen. (**C**) The mRNA expression levels of TNF-α was analyzed by RT-PCR in knee tissues from MVC-treated mice. (**D**,**E**) Representative dot plots of one mouse from each group. Normal control (NC) mice received 1% (*v*/*v*) DMSO in saline and MVC (50 mg/kg) intraperitoneally (ip) daily from days 21 to 35. CIA mice were treated with MVC (50 mg/kg) ip after the second immunization. The level of significance was set at * *p* < 0.05 compared with the CIA control group. Data are presented as mean ± SD (n = 6).

To further evaluate whether MVC could inhibit inflammatory cytokine production during arthritis, spleen and knee tissues were collected from CIA control and saline-treated mice to measure TNF-α and RANTES using flow cytometry and RT-PCR analyses. The TNF-α- and RANTES-producing CD8+ T cells in CIA control mice were significantly more abundant than in saline-treated mice, whereas MVC treatment of CIA mice potentially reduced CD8+TNF-α+ and CD8+RANTES+ production (Figure 5A,B). Furthermore, MVC treatment of CIA mice inhibited TNF-α mRNA expression compared with CIA control mice (Figure 5C). As shown in Figure 5D,E, the representative dot plots of one mouse from each group. Our results demonstrated that the decrease in inflammatory cytokine production and expression with MVC could contribute to controlling the inflammatory process and joint damage in CIA mice.

## 3. Discussion

RA is considered a typical autoimmune rheumatic disease. A prominent feature of the rheumatoid synovium is the distribution of T cell subsets within the rheumatoid synovial compartment. CD8+ T cells, which are sparsely distributed throughout the synovial tissue, have been associated with the pathogenesis of autoimmune disorders [25,26]. A previous study reported that CD8+ T cells contributed to inflammatory cytokine production, indicating the role of this T cell subset in RA [27]. Taken together, these studies suggested that CD8+ T cells have a significant influence on RA pathogenesis. CCR5 DNA-polymorphism influences the severity of RA [28]. CCR5 contributes chemotactic activity in the synovial fluid of RA patients [29]. The accumulation of CCR5^+^ T cells in the synovium of patients with RA suggests an important role in disease pathology [30]. The present study explored the effects of MVC on various inflammatory mediators and transcription factors signaling in CD8^+^ T cells. These inflammatory mediators and transcription factors are known to be important for the proliferation and progression of inflammation during RA. Our results showed that MVC effectively modifies the expression of inflammatory mediators and transcription factors to accelerate the joint inflammation repair process and to prevent infiltration of inflammatory cells into the damaged area. This is the first study to demonstrate that the CCR5 antagonist MVC is effective at preventing CIA in mice, as evidenced by significant decreases in mean arthritic scores and improved histological features in the CIA mouse model. The therapeutic benefits of MVC in CIA mice could be related to an anti-inflammatory effect that is mediated through downregulation of Th9/Th17 cells. Our results suggest that MVC administration may be a potential anti-arthritic agent with novel mechanisms of action.

IL-9 is important for T cell activation and differentiation in autoimmune inflammation. Previous results provided evidence of a critical role of IL-9 in triggering disease progression and proposed that targeting IL-9 could be a successful strategy to mitigate synovial inflammation in RA [31]. A previous study exposed the potential role of IL-9 and Th9 cells in RA pathogenesis, which is associated with the degree of synovial inflammatory infiltrate [8]. Another study reported that IL-9 is a mediator of Th17-driven inflammatory disease [32,33]. Previous results indicated that the transcription factor IRF4 was upregulated in RA patients [34]. Furthermore, one study revealed that IRF4 was highly expressed in the synovial fluid and synovium of RA patients [35]. It was further reported that IRF4 signaling plays a critical role in RA development [36]. GATA3, an important transcription factor, showed significantly decreased expression in an RA mouse model [37]. Our results indicated that MVC treatment in CIA significantly decreased IL-9-producing CD8+ T cells. The decline in mRNA and protein levels of IL-9 was also observed in MVC-treated mice, suggesting an anti-inflammatory mechanism of MVC. Moreover, we found that MVC treatment reduced IRF4 and increased GATA3 production; furthermore, the mRNA and protein expression levels of GATA3 were increased in the knee tissues of MVC-treated CIA mice. These results suggest that MVC administration regulates the balance of IRF4/GATA3 transcription factors in CIA mice and indicate that MVC treatment could play a therapeutic role by blocking IL-9 related signaling in CIA mice.

RA was previously believed to be a Th1 cell-associated inflammatory disease; however, much evidence has revealed that Th17 cells play a critical role in RA. A previous study demonstrated that the IL-17 cytokine level was significantly higher in synovial fluid of RA patients [38]. Significantly suppressed arthritis development was observed in IL-17-deficient mice [39]. A previous study confirmed that IL-17 strongly induced pro-inflammatory cytokine and chemokine production [40]. It has been reported that RORγt is crucial in RA development, and the attenuation of its expression could control excessive progression of RA [41]. Previous findings also indicated that RORγt overexpression induced greater IL-17 production in CIA mice [42]. Enhanced IL-21R expression was found in RA synovial fibroblasts and macrophages [43]. Previous studies also showed that IL-21R-deficient mice had reduced joint swelling and histological inflammation [44,45]. To elucidate the effects of MVC on Th17 cells in CIA mice, we administered MVC to CIA mice and found significantly inhibited production and expression of IL-17A and RORγT in the spleen and knee tissues, suggesting that MVC had profound effects on IL-17A and RORγT expression. Our results further demonstrated that MVC-treated CIA mice had decreased IL-17A- and RORγt-producing IL-21R+ levels in the spleen. These results suggested that MVC could inhibit Th17 cells through downregulating RORγt expression, indicating that this may decrease the severity of arthritis in CIA mice. Therefore, these results support our hypothesis that IL-17/RORγT signaling inhibition could suppress arthritis development in CIA mice.

It has been reported that TNF-α has a complex role in RA pathogenesis by inducing joint inflammation and pannus formation, leading to cartilage erosion and bone destruction [46]. Additionally, it was shown that overproduction of TNF-α is an essential element in the propagation of fibroblast-like synoviocytes in RA [47]. Moreover, it is reported that inhibition of TNF-α could improve RA development [48]. Furthermore, it was confirmed that an increased TNF-α level is associated with RA development [49]. It is reported that RANTES participates in RA pathogenesis through facilitating leukocyte infiltration and collagen degradation [50]. The increased RANTES expression activates T cells and fibroblast-like synoviocytes into synovium [51]. In this study, we found that TNF-α and RANTES production was significantly elevated in CIA control mice; however, production of both was decreased by treating CIA mice with MVC. Our results also revealed that MVC-treated CIA mice had decreased mRNA levels of TNF-α in knee tissues. Our results suggested the anti-inflammatory effects of MVC in CIA mice, which could play a potential role in RA treatment.

Fleishaker et al. reported that MVC failed to demonstrate efficacy in the patients with active RA in a randomized, double-blind placebo-controlled trial [52]. This study showed that MVC treatment did not ameliorate primary and secondary clinical end-points in RA patients and this was corroborated by lack of any significant effect on ACR responder rates, CRP, or DAS [52]. The authors in this study further opined that the lack of efficacy could be due to late therapeutic intervention of MVC in RA patients as they had 8 years of persistent disease activity. Finally, it was suggested in this study that an early therapeutic intervention at onset of the disease could be more effective in RA patients [52]. However, other studies have suggested that MVC could be beneficial in variety of immune-mediated disorders through a reduction in inflammatory parameters [53,54]. It was also reported that MVC caused reduction in the atherosclerotic progression by interfering with inflammatory cell recruitment into plaques in a mouse model of genetic dyslipidemia [55]. Further, MVC was suggested a potentially effective new strategy to prevent visceral acute graft versus host disease in humans [22]. Furthermore, a recent study has shown that MVC administration attenuates inflammation in a murine model of experimental autoimmune encephalitis by decreasing pro-inflammatory cytokines and increasing anti-inflammatory cytokines [56]. However, more human/animal studies are needed to explore full potential of MVC in different autoimmune/inflammatory diseases.

To the best of our knowledge, this is the first study to investigate the role of the CCR5 antagonist MVC in CIA mice. MVC administration improved the progression of arthritis in CIA mice. Investigation of the molecular mechanism showed that MVC suppresses inflammatory mediators and regulates Th9/Th17-related transcription factors in CIA mice. Therefore, our results suggest that modulation of transcription factor signaling by the CCR5 antagonist MVC could be useful as a potential therapeutic treatment for RA.

## 4. Material Methods

### 4.1. Animals

Male DBA/1J mice were obtained from Jackson Laboratories (Bar Harbor, ME, USA). All mice were used at 10–12 weeks old and maintained at the College of Pharmacy Animal Facilities of King Saud University. Mice were divided into four groups (n = 6): dimethyl sulfoxide (DMSO) in saline treatment only as the normal control (NC) group, the maraviroc treatment (NC + MVC) group, collagen-induced arthritis (CIA control) group, and CIA + MVC treatment group. All experimental procedures were approved by the Scientific Research Ethical Committee by King Saud University (Ethical Approval No: KSU-SE-19-63).

### 4.2. Induction of Experimental CIA and MVC Administration

For CIA, mice were immunized on day 1 with intradermal injection of 100 μg bovine type II collagen emulsified in complete Freund’s adjuvant (Sigma-Aldrich, St. Louis, MO, USA) at the base of the tail, 2–3 cm from the body. This was followed by a booster of 100 μg collagen emulsified in incomplete Freund’s adjuvant (IFA; Sigma-Aldrich) injected at the base of the tail near the primary injection site on day 21, as previously described [57,58]. Mice were intraperitoneally (ip) injected with MVC (50 mg/kg) daily from day 21 until day 35. The CIA and NC groups were administered DMSO in saline only. The MVC dose was selected based on the results of a previous study [24]. We did not observe any signs of toxicity or death.

### 4.3. Clinical Assessment of Arthritis

To determine the severity of arthritis, the animals were visually inspected by two blinded independent investigators, which allowed for daily monitoring of signs of arthritis. Briefly, the scores were assigned based on erythema, swelling, or loss of function present in each paw according to a macroscopic scoring system: 0 = no sign of edema or swelling, 1 = swelling and/or redness of the paw or one digit, 2 = slight edema and involvement of two joints, 3 = moderate edema and involvement of more than two joints, and 4 = edema and erythema from the ankle to the entire leg. The arthritis score for each mouse was expressed as the sum of the scores of four limbs.

### 4.4. Histological Assessment

Knee joints were removed and fixed in 10% neutral-buffered formalin. For standard hematoxylin and eosin staining, the fixed knee joints were decalcified in a 10% nitric acid solution in distilled water. Fresh solution was changed every other day until decalcification was completed. After decalcification, the knee joints were transferred to 70% ethanol for paraffin embedding and processing. Sections (7 μm) were stained with hematoxylin and eosin (Sigma-Aldrich) as described previously [26]. The slides were photographed and analyzed by a histopathologist.

### 4.5. Flow Cytometric Analysis

Flow cytometry analyses were performed to assess IL-21R, IL-9, IL-17A, IRF4, GATA3, RORγT, TNF-α, and RANTES production in CD8 T cells from the spleens of CIA mice. Briefly, splenocytes were stimulated for 4 h with ionomycin (Sigma-Aldrich) and phorbol 12-myristate 13-acetate in the presence of Golgi-Plug (BD Biosciences, San Jose, CA, USA), as described previously [59]. Cells were washed, and surface staining of CD8 and IL-21R cell surface receptors (BioLegend, San Diego, CA, USA) was performed. After fixation and permeabilization (BioLegend), cells were stained intracellularly with Th9 (anti-IL-9, anti-IRF4, and anti-GATA3; BioLegend), Th17 (anti-IL-21R, anti-IL-17A, and anti-RORγT; BioLegend), anti-TNF-α, and anti-RANTES (BioLegend) fluorescent antibodies. To determine different immune markers in the lymphocytes, lymphocytes were isolated from rest of the immune cells (monocytes and granulocytes) through conventional gating strategy which gates lymphocytes based on physical properties (forward and side scatter). Cytokines and transcription factors were then determined based on immunofluorescence characteristics of the antibody-labeled cells in the lymphocyte gate (supplementary Figure S1). Gating strategy for determination of percentages of CD8+IL-9+, CD8+IRF-4+, CD8+GATA3+, CD8+IL-17A+, CD8+RORγT+, IL-21R+IL-17A+, IL-21R+RORγT+, CD8+TNF-α+, and CD8+RANTES+ cells is shown in (Figure S1). Isotype controls were also run for each intracellular protein of interest. Viability of cells was also assessed using 7-AAD (Figure S1). The cells were analyzed on an FC500 flow cytometer (Beckman Coulter, Indianapolis, IN, USA). The events were collected and analyzed with CXP software (Beckman Coulter).

### 4.6. Real-Time RT-PCR

Total RNA was isolated from knee tissues using TRIzol reagent (Life Technologies, Carlsbad, CA, USA). Then, cDNAs were prepared and analyzed for expression of the gene of interest by quantitative real-time PCR (Real-Time PCR System, Applied Biosystems, Foster City, CA, USA) using a SYBR-Green PCR Master Mix Kit as previously described [60]. PCR primers used for IL-9, IRF4, TNF-α, IL-17A, RORγt, and GAPDH were purchased from Genscript (Piscataway, NJ, USA). The primers sequences were as follows: IL-9, F: 5′-GACCAGCTGCTTGTGTCTCT-3′ and R: 5′-GGACGGACACGTGATGTTCT-3′; IRF4, F: 5′-TGGAGGGATTATGCCCCTGA-3′ and R: 5′-AGCAGAGGTTCCACATGAGC-3′; TNF-α, F: 5′-ATGGCCTCCCTCTCATCAGT-3′ and R: 5′-ACCCTGAGCCATAATCCC CT-3′; IL-17A, F: 5′-GGACTCTCCACCGCAATGAA-3′ and R:5′-GGGTTTCTTAGGGG TCAGCC-3′; RORγt, F: 5′-AGCTGTGGGGTAGATGGGAT-3′ and R:5′-ATCCGGTC CTCTGCTTCTCT-3′; and GAPDH, F: 5′-TGATGGGTGTGAACCACGAG-3′ and R: 5′-AAGTCGCAGGAGACAACCTG-3′. The real-time PCR data were analyzed using the relative gene expression method. The samples were normalized to GAPDH.

### 4.7. Western Blot Analysis

Protein was extracted from knee tissues as previously described [61]. Protein quantitation was performed using the Direct Detect**^®^** Infrared Spectrometer (Merck, Darmstadt, Germany). Protein samples were separated by SDS gel electrophoresis and transferred to a nitrocellulose membrane (Bio-Rad, Hercules, CA, USA). Membranes were stained with primary antibodies to IL-9, IL-17A, RORγt, GATA3, and β-actin; then, HRP-conjugated secondary antibody was added (Santa Cruz Biotechnology, Santa Cruz, CA, USA). The bands corresponding to IL-9, IL-17A, RORγt, GATA3, and β-actin were visualized using a Western Blot Detection Chemiluminescence Kit (Merck) and quantified in relation to β-actin bands.

### 4.8. Statistical Analysis

Results are expressed as the mean ± SD. The significance of the results was analyzed using one-way ANOVA followed by Bonferroni’s post-hoc comparisons test using GraphPad Prism 5 software (GraphPad Software, La Jolla, CA, USA). *p*-values < 0.05 were considered significant.

## Data Availability

The authors confirm that all data underlying the findings are fully available without restriction. All relevant data are within the paper.

## Supplementary Materials

The supplementary materials are available online, Figure S1: immunofluorescence characteristics of the antibody-labeled cells in the lymphocyte gate.
