# Supplementary material for: Chemokine Receptor 5 Antagonism Causes Reduction in Joint Inflammation in a Collagen-Induced Arthritis Mouse Model
Source: Molecules. 2021 Mar 25;26(7):1839. doi: 10.3390/molecules26071839 (PMC8036613; doi:10.3390/molecules26071839)
Supplement: Supplementary file 1 [file molecules-26-01839-s001.pdf]

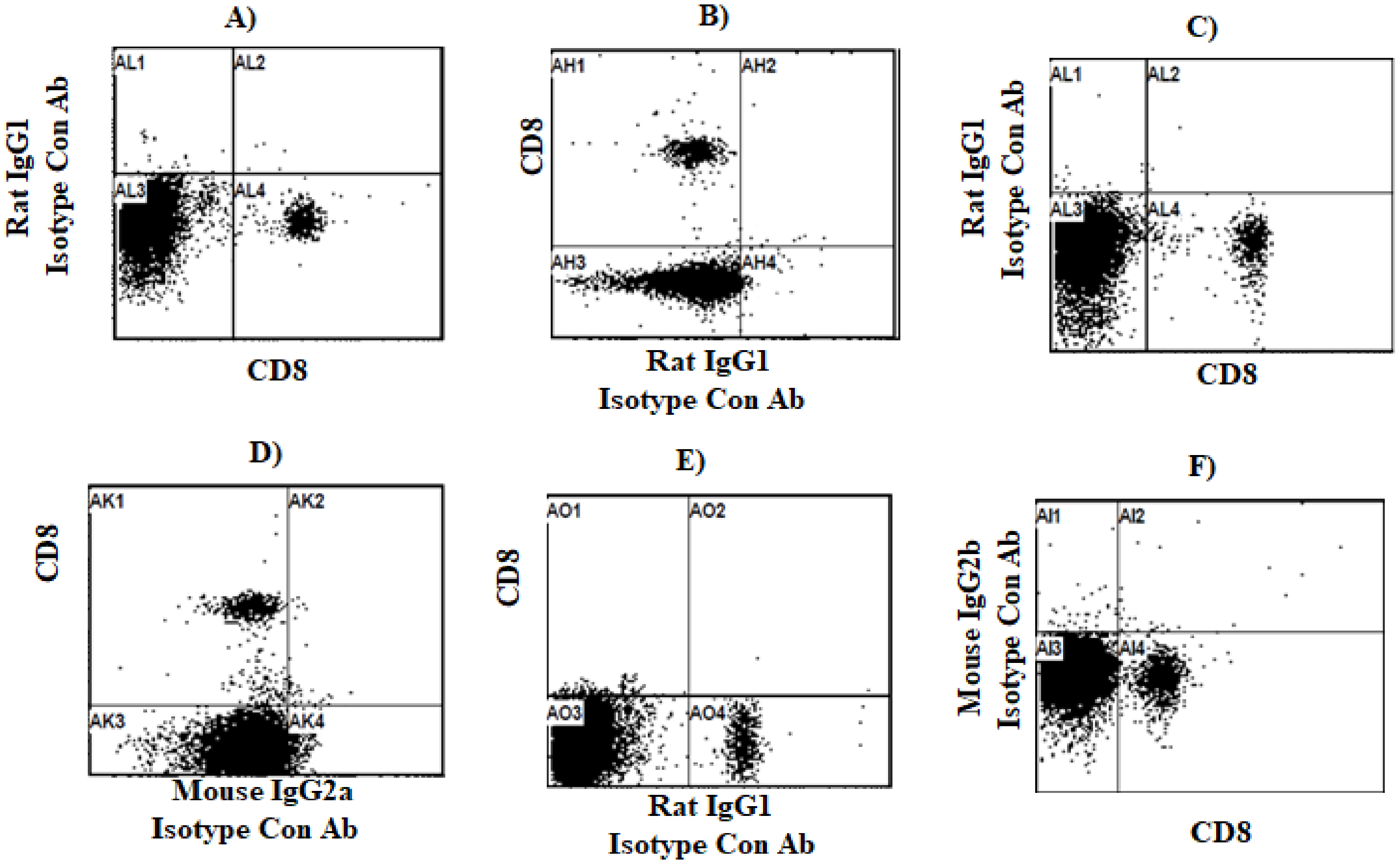


**Suppl Fig. 1**
A) Isotype control for Fig. 2D.
B) Isotype control for Fig. 3E.
C) Isotype control for Fig. 4I.
D) Isotype control for Fig. 4G.
E) Isotype control for Fig. 5D.
F) Isotype control for Fig. 5E.
There was almost negligible immunostaining for the proteins of interest on $CD8^+$ T cells with isotype control antibodies (i.e. in absence of protein specific antibodies).

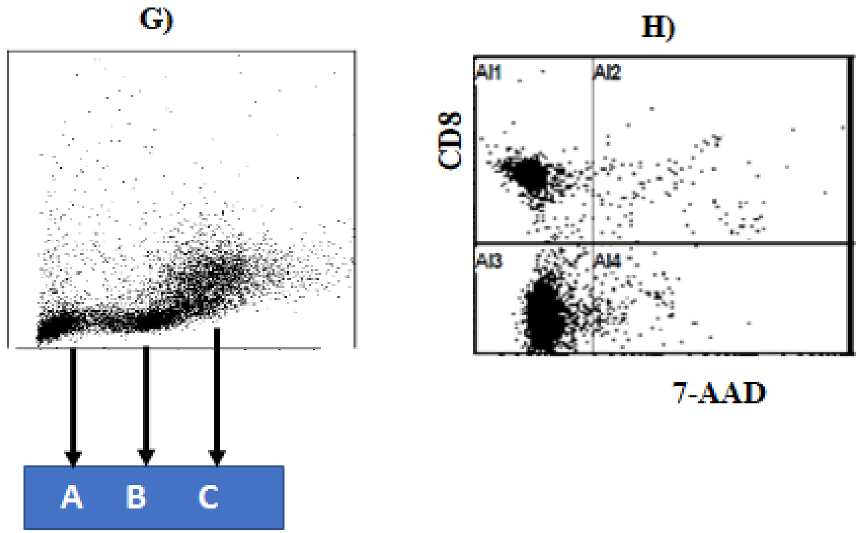


**Suppl Fig. 1**
G) Representative forward and side scatter plot of spleen cells. A, B, and C depict lymphocyte, monocyte and granulocyte gates respectively according to conventional gating strategy. Gate A was further selected for investigation of biomarkers on T cells.
H) 7-AAD staining on $CD8^+$ T cells in A gating. Most of the $CD8^+$ T cells were viable as there were <5% dead cells in $CD8^+$ T compartment, i.e. cells showing double positive immunostaining for both 7-AAD and $CD8^+$ T cells (quadrant AI2).
